# Biosynthesis of compatible solutes in rhizobial strains isolated from *Phaseolus vulgaris *nodules in Tunisian fields

**DOI:** 10.1186/1471-2180-10-192

**Published:** 2010-07-16

**Authors:** Cristina Fernandez-Aunión, Thouraya Ben Hamouda, Fernando Iglesias-Guerra, Montserrat Argandoña, Mercedes Reina-Bueno, Joaquín J Nieto, M Elarbi Aouani, Carmen Vargas

**Affiliations:** 1 Department of Microbiology and Parasitology, University of Seville, Spain; 2 Laboratory of Legumes. Centre of Biotechnology of Borj Cedria, BP 901 Hammam-lif 2050, Tunisia; 3 Department of Organic and Pharmaceutical Chemistry, University of Seville, Spain; 4 Current Address: NEPAD/North Africa Biosciences Network. National Research Center, El Buhouth St, Dokki, Cairo, 12311 Egypt

## Abstract

**Background:**

Associated with appropriate crop and soil management, inoculation of legumes with microbial biofertilizers can improve food legume yield and soil fertility and reduce pollution by inorganic fertilizers. Rhizospheric bacteria are subjected to osmotic stress imposed by drought and/or NaCl, two abiotic constraints frequently found in semi-arid lands. Osmostress response in bacteria involves the accumulation of small organic compounds called compatible solutes. Whereas most studies on rhizobial osmoadaptation have focussed on the model species *Sinorhizobium meliloti*, little is known on the osmoadaptive mechanisms used by native rhizobia, which are good sources of inoculants. In this work, we investigated the synthesis and accumulations of compatible solutes by four rhizobial strains isolated from root nodules of *Phaseolus vulgaris *in Tunisia, as well as by the reference strain *Rhizobium tropici *CIAT 899^T^.

**Results:**

The most NaCl-tolerant strain was *A. tumefaciens *10c2, followed (in decreasing order) by *R. tropici *CIAT 899, *R. leguminosarum *bv. *phaseoli *31c3, *R. etli *12a3 and *R. gallicum *bv. *phaseoli *8a3. ^13^C- and ^1^H-NMR analyses showed that all *Rhizobium *strains synthesized trehalose whereas *A. tumefaciens *10c2 synthesized mannosucrose. Glutamate synthesis was also observed in *R. tropici *CIAT 899, *R. leguminosarum *bv. *phaseoli *31c3 and *A. tumefaciens *10c2. When added as a carbon source, mannitol was also accumulated by all strains. Accumulation of trehalose in *R. tropici *CIAT 899 and of mannosucrose in *A. tumefaciens *10c2 was osmoregulated, suggesting their involvement in osmotolerance. The phylogenetic analysis of the *otsA *gene, encoding the trehalose-6-phosphate synthase, suggested the existence of lateral transfer events. *In vivo *^13^C labeling experiments together with genomic analysis led us to propose the uptake and conversion pathways of different carbon sources into trehalose. Collaterally, the β-1,2-cyclic glucan from *R. tropici *CIAT 899 was co-extracted with the cytoplasmic compatible solutes and its chemical structure was determined.

**Conclusions:**

The soil bacteria analyzed in this work accumulated mainly disaccharides in response to NaCl stress. We could not find a direct correlation between the trehalose content of the rhizobial strains and their osmotolerance, suggesting that additional osmoadaptive mechanism should be operating in the most NaCl-tolerant strain *R. tropici *CIAT 899.

## Background

*Rhizobium*-legume symbiosis represents the most important nitrogen-fixing mechanism, which may have the potential to increase nitrogen input in arid and semi-arid ecosystems. However, biotic (i.e., pests or diseases), and abiotic (i.e., salinity, drought, high temperature or heavy metals) constraints limit legume crop production in arid and semi-arid lands, which are often located in developing countries [1]. Both drought and salinity impose osmotic stress, as a result of large concentrations of either salt or non-ionic solutes in the surrounding medium, with the resulting deficit of water [2]. The *Rhizobium*-legume symbiosis is highly sensitive to osmotic stress. Therefore strategies to improve the symbiosis efficiency and legume production under this constraint should target both symbiotic partners, together with appropriate crop and soil management [1].

Rhizospheric rhizobia are subjected to frequent fluctuations in the osmolarity of their environment due to the succession of drought and rain periods, the exclusion of salts like NaCl from root tissues, the release of plant exudates, or the production of exopolymers by plant roots and rhizobacteria. In addition, rhizobia must also adapt to the osmotic situation during the infection process and in a nodule exchanging nutrients with the host plant [3]. Therefore, besides symbiotic efficiency, osmotolerance may constitute a competitive trait for either native or inoculant rhizobia, in order to persist in drought/salt-affected soils, and/or after the process of seed coat-mediated desiccation, and maybe to improve the colonization and/or infection process.

One of the main mechanisms of bacterial adaptation to hyperosmotic conditions is the intracytoplasmic accumulation of low molecular-weight organic osmolytes [2,4]. These molecules are termed compatible solutes because they do not interact with macromolecules in detrimental ways [5]. Compatible solutes are accumulated either by uptake from the environment (exogenous compatible solutes or osmoprotectants) or by *de novo *biosynthesis (endogenous compatible solutes). The diversity of compatible solutes is large but falls into a few major chemical categories, such as sugars (i.e., sucrose, trehalose), polyols (i.e,, sorbitol, mannitol), amino acids and derivatives (i.e. proline, glutamate, glutamine), betaines and ectoines [4]. It is very common for microorganisms to use a cocktail of compatible solutes, a strategy that allows the cell to adapt the compatible solute pool to different environmental injuries. Indeed, the role of compatible solutes goes beyond osmotic adjustment alone, to protection of cells and cell components from freezing, desiccation, high temperature and oxygen radicals [4,6,7]. On the other hand, hypoosmotic adaptation in gram-negative bacteria, including the *Rhizobiaceae*, involves the accumulation of periplasmic cyclic glucans, which appear to contribute substantially to periplasmic osmolarity [3,8].

Among the *Rhizobiaceae*, the best studied species regarding osmoadaptation is *Sinorhizobium meliloti *one of the most common alfalfa microsymbionts. Specific concomitant accumulation of potassium and glutamate was found to be the primary response in *S. meliloti *to hyperosmotic stress [9]. Out of four potassium uptake systems found within the *S. meliloti *genome, Trk was shown to be the most important K^+ ^importer involved in the osmoadaptation of this bacterium [10]. By using ^13^C nuclear magnetic resonance spectroscopy (a particularly useful technique for osmoadaptation studies because all types of organic compounds can be detected at once), it was shown that *S. meliloti *long term response to hyperosmotic stress involves the synthesis and accumulation of the dipeptide N-acetylglutaminylglutamine amide and the disaccharide trehalose, the latter one specially when cells are subjected to severe osmotic stress [3,11].

Trehalose is a non-reducing glucose disaccharide that is widespread in nature. It protects numerous biological structures against abiotic stresses including desiccation, oxidation, heat, cold, dehydration, and hyperosmotic conditions [6]. Recently, the importance of trehalose in osmotolerance and nodulation of their legume hosts by *S. meliloti *[12] and *Bradyrhizobium japonicum *[13] has been firmly established. Trehalose has shown to play also a major role in desiccation tolerance of *R. leguminosarum *bv. *trifolii *[14].

Common bean (*Phaseolus vulgaris*) is an important staple crop in the diets of people of Latin America, Asia, Africa, and other regions of the developing world. Paradoxically, despite common bean is a promiscuous legume able to form symbioses with a number of rhizobial species including *R. tropici*, *R. etli*, *R. gallicum*, *R. leguminosarum bv. phaseoli *or *R. giardinii *[15-17], it is considered as a poor nitrogen fixer, if compared to other grain legumes [18,19]. This problem has been attributed to the ineffectiveness of indigenous rhizobia [20] or to adverse abiotic conditions [21]. In a recent work, Suarez et al. [22] reported an increase in root nodule number and nitrogen fixation by *P. vulgaris *cv. Negro Jamapa (a Mesoamerican cultivar) inoculated with a trehalose-6-phosphate synthase-overexpressing strain of *R. etli*. Thus, manipulating trehalose metabolism in *P. vulgaris *looks a promising strategy to improve plant tolerance to osmotic stress and grain yield. Compared to this body of knowledge on the osmoadaptation of these agronomically important rhizobacteria, little is known about the osmostress responses of rhizobial strains nodulating common bean in Africa. The purpose of the work described here was threefold. First, we used ^13^C-NMR to compare the osmoadaptive responses of three effective *Rhizobium *strains isolated from root nodules of *P vulgaris *grown in Tunisian fields under rainfed conditions [23,24] with that of the reference strain *R. tropici *CIAT 899^T^, a Latin American isolate that has been shown to tolerate several abiotic stresses, including high temperature, low pH, or salinity [15,25,26]. Despite a number of *R. tropici *CIAT 899 osmosensitive mutants has been characterized, none of them was affected in compatible solute synthesis [26,27]. In fact, the complete set of compatible solutes in this strain was unknown previously to this work. Second, we aimed to determine the osmoadaptive mechanism of *Agrobacterium *sp. 10c2 (proposed in this paper as *A. tumefaciens *10c2), which was isolated from the same Tunisian common bean fields as the above strains [24]. *Agrobacterium *sp. 10c2 could not nodulate *P. vulgaris per se*, but it was able to colonize pre-formed *P. vulgaris *nodules [28] and to modulate, either positively or negatively, nodulation of common beans by native rhizobia [29]. Third, we focused on trehalose, which we found as the major compatible solute in the four *Rhizobium *strains. We determined the trehalose content of the strains and traced its biosynthetic pathway both molecularly and biochemically. Collaterally, the β-1,2-cyclic glucan from *R. tropici *CIAT 899 was co-extracted with the cytoplasmic compatible solutes when cells were grown at low salinity, and its chemical structure was determined by using a suite of one-dimensional and two-dimensional NMR spectra and mass spectrometry.

## Results

### Strain identity and phylogeny

**Figure 1:**
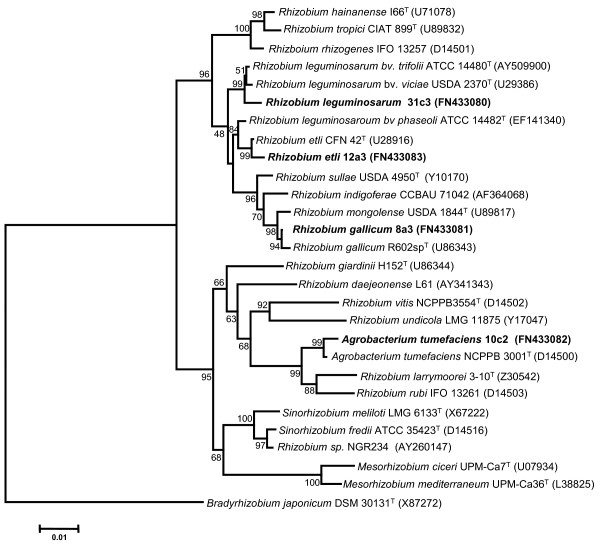
**Identity and phylogeny of rhizobial strains isolated from common bean nodules**. Neighbor-joining tree based on 16S rDNA sequences of the isolates and other related species of the family *Rhizobiaceae*. The tree is drawn to scale, with branch lengths in the same units as those of the evolutionary distances used to infer the phylogenetic tree. All positions containing alignment gaps and missing data were eliminated only in pairwise sequence comparisons. Bootstrap probabilities (as percentage) were determined from 1000 resamplings. Bar, 0.01 substitutions per nucleotide position. Note that the validated name of the genus *Sinorhizobium *is *Ensifer *[59].

Strains *R. gallicum *bv. *gallicum *8a3, *R. etli *12a3, *Agrobacterium *sp. 10c2 and *R. leguminosarum *bv. *phaseoli *31c3 were previously isolated by Mhamdi et al. [23] from nodules of *P. vulgaris *grown on neutral soil samples collected from North Tunisia. A preliminary strain affiliation was made upon RFLP analysis of the 16S rRNA, *nodC *and *nifH *genes [24], and partial sequence of the 16S rDNA and BLAST search for homologous sequences (for *Agrobacterium *sp. 10c2 [28]). To confirm the identity and phylogenetic position of the strains, we sequenced their nearly complete 16S rDNA Figure 1 shows the phylogenetic tree constructed using the neighbor-joining method based on these sequences and those of closely related rhizobia obtained from GeneBank. Strains *R. etli *12a3, *R. gallicum *bv. *phaseoli *8a3, and *Agrobacterium *sp. 10c2 grouped with the *R. etli*, *R. gallicum *and *A. tumefaciens *type strains. On the basis of its phylogenetic relatedness to the type strain of *A. tumefaciens*, we propose strain *Agrobacterium *sp. 10c2 to be named as *A. tumefaciens *10c2. *R. leguminosarum *bv. *phaseoli *31c3 was in the same cluster as the type strains of *R. leguminosarum *bvs. *trifolii *and *viciae*, but in a separate branch. Interestingly, the type strain of *R. leguminosarum *bv. *phaseoli*, was in a separate group, close to the *R. etli *type strain. This lack of clustering between the type strains of *R. leguminosarum *bv. *phaseoli *and the other two biovars of *R. leguminosarum *was previously reported [30], and it was proposed that *R. phaseoli *should be retained as a separate species.

### Differences in halotolerance of the strains

**Figure 2:**
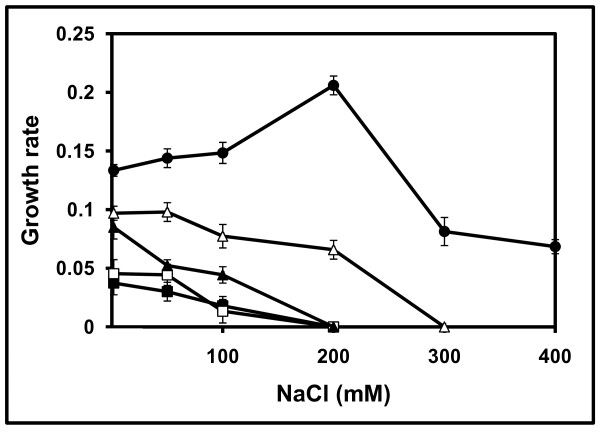
**Effect of NaCl concentration on the growth rates of the strains isolated from common bean nodules**. *R. gallicum *8a3 (■) was grown in M79-I with 20 mM glucosa. *R. etli *12a3 (□) and *R. leguminosarum *31c3 (▲) were grown in M79-I with 20 mM mannitol, and *R. tropici *CIAT 899 (control) (Δ) and *A. tumefaciens *10c2 (●) were grown in MAS with 20 mM mannitol. Growth rates are expressed as ΔOD_600_/h. Values shown are the mean of two replicas of each condition in three independent experiments ± SD (standard deviation).

As a previous step to investigate the compatible solute content of the local and reference strains, we selected a suitable minimal medium for their growth and determined their tolerance to NaCl. Growth was tested in two chemically defined minimal media (M79-I or MAS) with two different carbon sources (20 mM glucose or mannitol). Cell yield (as measured by turbidimetry) showed that *R. gallicum *bv. *phaseoli *8a3 grew better in M79-I with glucose, *R. leguminosarum *bv. *phaseoli *31c3 and *R. etli *12a3 in M79-I with mannitol, and *R. tropici *CIAT 899 and *A. tumefaciens *10c2 in MAS with mannitol (data not shown). Subsequently, cells were grown in 50 ml of their optimal minimal media containing increasing NaCl concentrations up to 600 mM NaCl. Cultures were incubated at 28°C and growth was monitored up to the stationary phase. The most salt-tolerant strain was *A. tumefaciens *10c2, which grew well in MAS medium containing 400 mM NaCl and displayed optimal growth at 200 mM NaCl (Figure 2). *A. tumefaciens *10c2 growth was totally impaired at 600 mM NaCl (not shown). The second most NaCl-tolerant strain was *R. tropici *CIAT 899, which grew well in MAS medium containing up to 200 mM NaCl and showed optimal growth at 50 mM NaCl. Strains *R. leguminosarum *bv. *phaseoli *31c3, *R. etli *12a3 and *R. gallicum *bv. *phaseoli *8a3 were the most salt sensitive ones, showing optimal growth in basal M79-I medium with no extra salt added. *R. leguminosarum *bv. *phaseoli *31c3 was slightly more salt-tolerant than *R. etli *12a3 and *R. gallicum *bv. *phaseoli *8a3. Growth of these three strains was severely impaired over 100 mM NaCl (Figure 2).

### Analysis of major intracellular solutes

**Figure 3:**
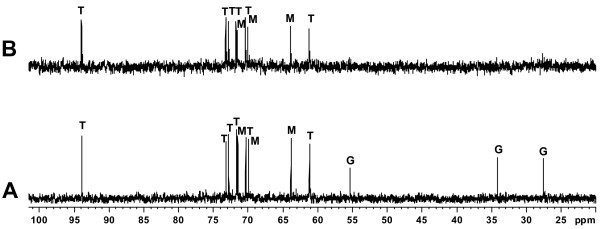
**Analysis of major intracellular solutes in *R. leguminosarum *bv. *phaseoli *31c3 and *R. etli *12a3**. *R. leguminosarum *bv. *phaseoli *31c3 (A) and *R. etli *12a3 (B) cells were grown in M79-I medium containing 0.1 M NaCl and 20 mM mannitol, and cellular extracts were analyzed by ^13^C-NMR. Resonances due to trehalose (T), mannitol (M), and glutamate (G) are indicated. Peaks due to the carboxylate groups of glutamate (at 175.2 and 181.9 ppm) are not shown.

The differences found in salt tolerance of the strains prompted us to investigate and compare their compatible solute content. For this purpose, we analyzed cellular extracts by using ^1^H- and ^13^C-NMR. The ^13^C-NMR spectrum of *R. leguminosarum *bv. *phaseoli *31c3 grown in mannitol M79-I medium with 100 mM NaCl contained three sets of chemical shifts that were assigned to the disaccharide trehalose (61.2, 70.4, 71.7, 72.8, 73.2, and 93.9 ppm), the sugar alcohol mannitol (63.9, 70.0, and 71.6 ppm) and the amino acid glutamate (27.6, 34.2, 55.4, 175.2, and 181.9 ppm) (Figure 3A). Trehalose and mannitol, but not glutamate, were also majoritarily found in extracts from strain *R. etli *12a3 cultivated in mannitol M79-I medium with 100 mM NaCl (Figure 3B). The identity of these three compatible solutes was confirmed by ^1^H-NMR analysis of extracts from the two strains (not shown).

**Figure 4:**
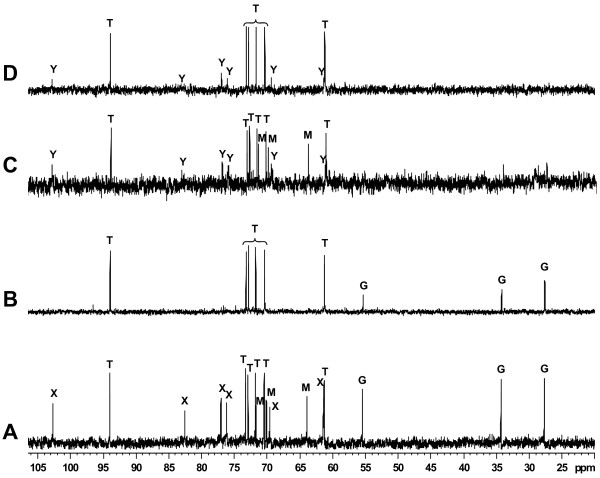
**Analysis of major intracellular solutes in *R. tropici *CIAT 899 and *R. gallicum *bv. *phaseoli *8a3**. *R. tropici *CIAT899 was grown in MAS minimal medium with 20 mM mannitol and 100 mM **(A) **or 200 mM **(B) **NaCl. *R. gallicum *bv. *phaseoli *8a3 was grown in M79-I minimal medium with 100 mM NaCl and 20 mM mannitol **(C) **or glucose **(D)**. Cellular extracts were analyzed by ^13^C-NMR. Resonances due to trehalose (T), mannitol (M), glutamate (G), and unknown sugars (X, Y) are indicated.

When grown in MAS medium with 100 mM NaCl in the presence of mannitol, *R. tropici *CIAT 899 spectrum displayed three sets of resonances that could be assigned to trehalose, mannitol and glutamate, and a fourth set of six sugar carbon resonances (at 61.3, 69.5, 76.1, 77.0, 82.5, and 102.6 ppm) that could not be initially assigned to any known compound (Figure 4A). However, the presence of a signal with a chemical shift above 102 ppm, indicated β configuration of a glucose unit. When the salt concentration was raised up to 200 mM NaCl in the same medium, only chemical shifts due trehalose and glutamate were observed, whereas those corresponding to mannitol and the unknown sugar were not detected (Figure 4B). Trehalose, mannitol, and an unknown minoritary sugar showing a similar resonance pattern as the unidentified compound found in *R. tropici *CIAT 899, were detected in the ^13^C-NMR spectra of *R. gallicum *bv. *phaseoli *8a3 grown in M79-I medium with 100 mM NaCl and mannitol (Figure 4C). However, mannitol was not accumulated in *R. gallicum *bv. *phaseoli *8a3 cultivated in the same medium with glucose as a carbon source (Figure 4D), suggesting that mannitol accumulation depends on its transport, rather than synthesis, in this strain.

**Figure 5:**
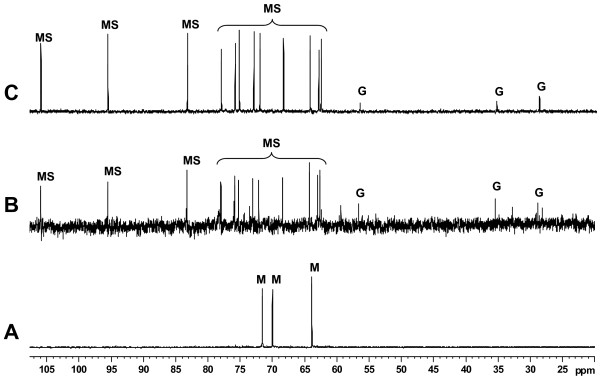
**Analysis of major intracellular solutes in *A. tumefaciens *10c2**. Cells were grown in MAS minimal medium with 20 mM mannitol and 100 mM (A), 200 mM (B) or 400 mM (C) NaCl. Cellular extracts were analyzed by ^13^C-NMR. Resonances due to trehalose (T), mannitol (M), glutamate (G), and mannosucrose (MS) are indicated. Peaks due to the carboxylate groups of glutamate (at 175.2 and 181.9 ppm) are not shown.

Figure 5 shows the ^13^C-NMR analysis of *A. tumefaciens *10c2 grown in mannitol MAS medium with increasing salinity. At the lowest salt concentration tested (100 mM NaCl), mannitol was the only intracellular solute detected (Figure 5A). However, above 100 mM NaCl mannitol was absent, and spectra contained five resonances attributed to glutamate and twelve resonances corresponding to the disaccharide mannosucrose (β-fructofuranosyl-α-mannopyranoside: 63.9, 64.3, 65.6, 69.7, 73.4, 74.3, 76.5, 77.2, 79.3, 84.6, 96.8, and 107.2 ppm) (Figures 5B and 5C). Identification of the latter was performed by comparison of the observed and published chemical shifts of this compound, which was reported to be accumulated by *A. tumefaciens *strain NT1 [31].

### Trehalose content of the rhizobial strains

**Figure 6:**
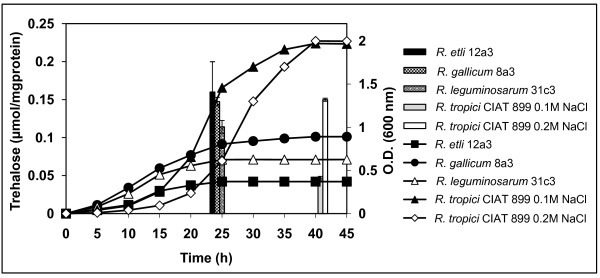
**Trehalose accumulation by *R. etli *12a3, *R. gallicum *bv. *phaseoli *8a3, *R. tropici *CIAT 899, and *R. leguminosarum *bv. *phaseoli *31c3**. Cells were grown in their optimal minimal medium up to early stationary phase, and trehalose content was measured colorimetrically as described in Methods. For each strain, a growth curve under the same condition used to measure trehalose accumulation is shown. Histograms representing trehalose accumulation are place above the sampling time. Trehalose values shown are the mean of three replicas of each condition in two independent experiments ± SD (Standard deviation).

As the four *Rhizobium *strains which accumulated trehalose displayed different salt tolerance, we investigated if there was a correlation between their intracellular trehalose content and their tolerance to salinity. For this purpose, trehalose was quantified colorimetrically from cells grown up to early stationary phase in their optimal minimal medium with 0.1 M (all strains) or 0.2 M NaCl (only CIAT 899) NaCl. As illustrated in Figure 6, intracellular trehalose content of strains *R. leguminosarum *bv. *phaseoli *31c3, *R. etli *12a3 and *R. gallicum *bv. *phaseoli *8a3 grown at 0.1 M NaCl ranged from 0.11 to 0.16 μmol/mg protein. At the same salinity, cells of the more salt-tolerant *R. tropici *CIAT 899 accumulated ca. 0.03 μmol of trehalose per mg of protein, but they displayed a 3.2-fold higher trehalose content when they were grown at 0.2 M NaCl, suggesting that trehalose accumulation in this strain is osmoregulated. However, even at 0.2 M NaCl trehalose levels of *R. tropici *CIAT 899 were equivalent to those of the more salt-sensitive strains *R. leguminosarum *bv. *phaseoli *31c3 and *R. gallicum *bv. *phaseoli *8a3 grown under their NaCl limiting conditions (0.1 M NaCl). The above data suggest that there is not a direct correlation between trehalose content of the strains and their salt tolerance. In addition, they suggest that, although trehalose accumulation in *R. tropici *CIAT 899 is osmoregulated, trehalose alone cannot account for the higher halotolerance of *R. tropici *CIAT 899.

### Isolation and phylogenetic analysis of the *otsA *gene

Since all *Rhizobium *strains tested synthesized trehalose, we were interested to check if this occurs through the OtsA-OtsB pathway. This very well conserved route involves the transfer of glucose from UDP-glucose to glucose-phosphate to form trehalose-6-phosphate by trehalose-6-phosphate synthase (OtsA). Then, a trehalose-6-phosphate phosphatase (OtsB) dephosphorylates this intermediate to produce trehalose [32,33]. The *otsA *genes of *R. leguminosarum *bv. *trifolii *[14], *S. meliloti *[12], *R. etli *[22] and *B. japonicum *[13] have been recently isolated. To check the presence of *otsA *in the genome of the *Rhizobium *strains, we designed oligonucleotides covering two very well-conserved regions and amplified the corresponding genes from genomic DNA of the selected strains. Single PCR products of ca. 1 kb were obtained from genomic DNAs of *R. etli *12a3, *R. gallicum *bv. *phaseoli *8a3 and *R. leguminosarum *bv. *phaseoli *31c3 (by using the primers OTA1 and OTA2), and *R. tropici *CIAT 899 (by using the primers OTAS1 and OTAS2). As expected, *A. tumefaciens *10c2 DNA was not amplified with any of the two *otsA *primer pairs.

**Figure 7:**
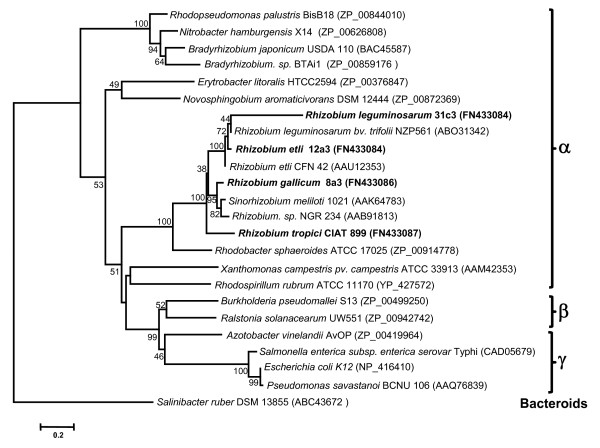
**Neighbor-joining tree based on OtsA proteins from α-, β-, and γ -proteobacteria**. The tree is drawn to scale, with branch lengths in the same units as those of the evolutionary distances used to infer the phylogenetic tree. The Bacteroides/Chlorobi representative *S. ruber *was used as outgroup. The evolutionary distances were computed using the Poisson correction method and are in the units of the number of amino acid substitutions per site. The rate variation among sites was modeled with a gamma distribution (shape parameter = 1). All positions containing gaps and missing data were eliminated from the dataset (complete deletion option). There were a total of 287 positions in the final dataset. Bootstrap probabilities (as percentage) were determined from 1000 resamplings and values less than 30 have been omitted.

The aligned OtsA proteins were subjected to phylogenetic analysis, and the resulting tree is shown in Figure 7. As expected, the OtsA proteins from *R. tropici *CIAT 899, *R. etli *12a3, *R. gallicum *bv. *phaseoli *8a3 and *R. leguminosarum *bv. *phaseoli *31c3 grouped with OtsA proteins of α-proteobacteria, but some incongruencies were found. For example, *R. gallicum *bv. *phaseoli *8a3 OtsA was more related to the OtsA proteins of *Sinorhizobium *(i.e. *S. meliloti *1021 or *Rhizobium *sp. NGR 234) than to those of *R. etli *or *R. leguminosarum*. In addition, *R. etli *12a3 OtsA did not cluster with *R. etli *CFN 42 OtsA but with the OtsA proteins from *R. leguminosarum *bv. *phaseoli *31c3 and *R. leguminosarum *bv. *trifolii*. From the above results, we suggest that the OtsA-OtsB pathway may be involved in trehalose synthesis in all strains tested.

### Synthesis of trehalose by *R. tropici *CIAT 899 from different carbon sources

**Figure 8:**
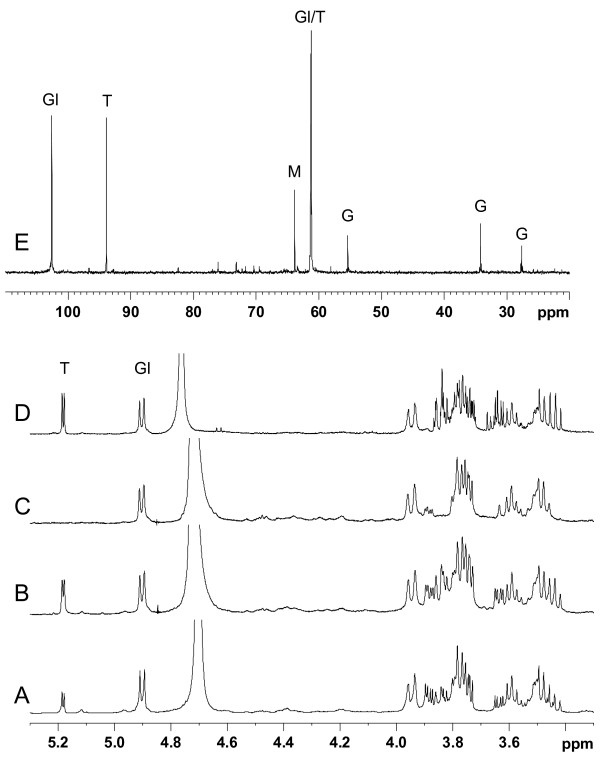
**Synthesis of trehalose by *R. tropici *CIAT 899 from different carbon sources**. ^1^H-NMR analysis of cellular extracts from *R. tropici *CIAT899 grown in 100 mM NaCl MAS medium containing glucose **(A)**, galactose **(B)**, mannose **(C) **or manitol **(D) **as a carbon source. T and Gl indicate the signals corresponding to the anomeric protons of the glucose units of trehalose and the cyclic glucan, respectively. **(E) **^13^C-NMR spectra of intracellular solutes accumulated by *R. tropici *CIAT899 grown in 0.1 M NaCl MAS medium with ^13^C1/6 manitol as a carbon source. Abbreviations: T, trehalose; Gl, cyclic β-glucan; M, manitol; G, glutamate.

The results presented so far indicated that trehalose is synthesized from mannitol-derived glucose via the OtsA-OtsB pathway in the four *Rhizobium *strains tested. We were interested to know if trehalose could be also synthesized from other carbon sources. For this purpose, *R. tropici *CIAT 899 was grown in 0.1 M NaCl MAS with glucose, galactose, mannose and mannitol and the accumulated compounds were analyzed by ^1^H NMR. Figure 8A-D shows that whereas the unknown sugar (later identified as a cyclic β-glucan) was synthesized from any of the tested carbon sources, trehalose was only accumulated when glucose, galactose or mannitol, but not mannose, was present in the culture medium.

To elucidate if the synthesis of trehalose by *R. tropici *CIAT 899 involves the transformation of mannitol to one or both of the trehalose glucose units, or a full degradation of the carbon source followed by a synthesis *de novo*, this strain was grown in 0.1 M NaCl MAS medium with 1-^13^C-mannitol as carbon source, and the cellular extracts were analyzed by ^1^H spectroscopy. As shown in Figure 8E, only resonances corresponding to the C1 and C6 carbons of the glucose units of trehalose and the unknown sugar, as well as those of the C1/C6 of mannitol, could be observed. In contrast, the three signals corresponding to glutamate were ^13^C-labelled. These findings indicate that the two glucose moieties of trehalose, as well as the unknown sugar units, were derived directly from mannitol, whereas glutamate synthesis occurred *de novo*, after complete mannitol degradation.

### The unknown sugar accumulated by *R. tropici *CIAT 899 at low salinity is a cyclic (1→2)-β-glucan

**Figure 9:**
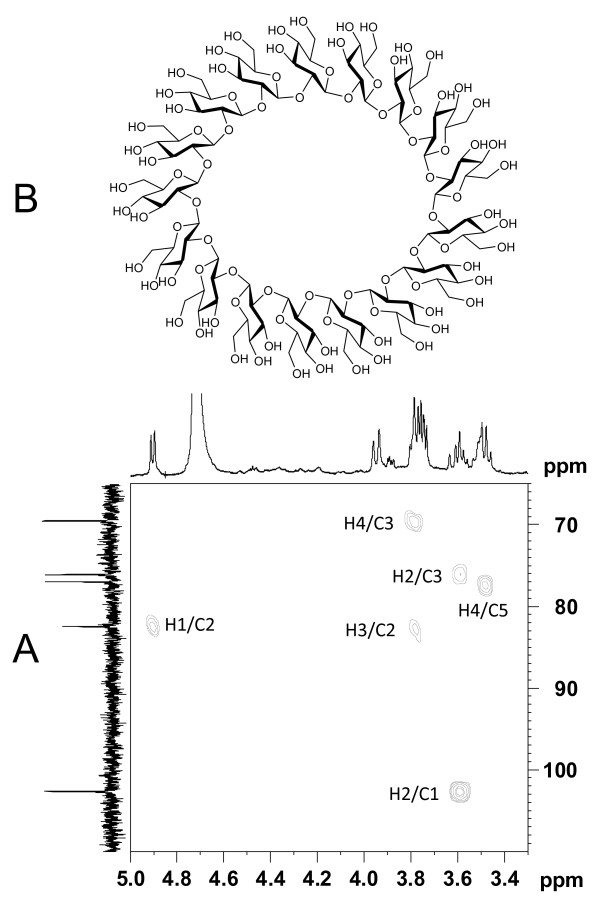
**Identification of the *R. tropici *CIAT899 cyclic (1→2)-β-glucan**. (A) HMBC spectrum of intracellular solutes accumulated by *R. tropici *CIAT899 grown in MAS medium with mannose and 100 mM NaCl. (B) Chemical structure of the cyclic (1→2)-β-glucan.

Initially, the six remaining resonances in the ^13^C-NMR spectrum of cellular extracts from *R. tropici *CIAT 899 grown at low salinity could not be assigned to any known compatible solute (see Figure 3A). To determine the structure of this unknown sugar, we took advantage of the fact that *R. tropici *grown in the presence of mannose does not synthesize trehalose, which could interfere in the identification of this compound. Thus, cells of *R. tropici *were grown in MAS medium containing 20 mM mannose as a carbon source, and the unknown sugar was identified by using a set of ^1^H-NMR, COSY, NOESY, HSQC, HMBC and MS experiments. First, in the ^1^H spectrum, a doublet at 4.87 ppm (*J *7.9 Hz) was unequivocally assigned to the anomeric hydrogen of a β-glycoside unit. Second, the combination of the COSY and NOESY spectra (not shown) and the ^1^H-^13^C HSQC spectrum permitted the assignment of all proton and carbon signals in the compound (Table 1). Third, the HMBC experiment confirmed a 1→2 link between two monosaccharide, unsubstituted, molecules (Figure 9A). Finally, the mass spectrum showed a peak at *m/z *1400 corresponding to [M+Na]^++^, from which we could deduce a molecular weight of 2754, corresponding to 17 β-glucopyranose units. On the basis of this result, the structure of the compound was established as a cyclic (1→2)-β-glucan formed by 17 β-glucopyranose units (Figure 9B). This compound had been previously described as an extracellular glucan secreted by *R. tropici *CIAT 899 [34]. Our results clearly indicate that, as expected, the *R. tropici *CIAT 899 cyclic (1→2)-β glucan is also cell-associated.

**Table 1:** ^1^H and ^13^C NMR data (δ, ppm) for the *R. tropici *CIAT 899 cyclic (1→2)-β-glucan

	1	2	3	4	5	6
**H**	4.87	3.59	3.79	3.48	3.52	3.95, 3.74
**C**	102.6	82.5	76.1	69.5	77.0	61.3

^a ^^1^H and ^13^C signals were referenced to internal tetramethylsilane.

## Discussion

In this work, we investigated the osmoadaptive mechanisms used by four native rhizobia isolated from root nodules of *P. vulgaris *cultivated in north Tunisia [23]. Strains *R. etli *12a3, *R. gallicum *bv. *phaseoli *8a3 and *R. leguminsarum *31c3 are potentially good inoculants as they were infective and showed efficient nitrogen fixation in symbiosis with *P. vulgaris *[23]. In addition, *Agrobacterium *10c2 was able to colonize preformed *P. vulgaris *nodules [28] and to specifically favour nodulation by some local strains [29], suggesting that it might be used as co-inoculant. Our results confirm the strain affiliations proposed by Mhandi et al. [24,28]. In addition, on the basis of its phylogenetic relatedness to the *A. tumefaciens *type strain, *Agrobacterium *10c2 is proposed in this work to be renamed as *A. tumefaciens *10c2.

As shown by ^13^C- and ^1^H-NMR analyses, the long-term response of the four *Rhizobium *strains to NaCl involved the accumulation of trehalose, mannitol and glutamate; but the latter one was only observed in *R. leguminsarum *31c3 and *R. tropici *CIAT 899. The reason why glutamate was not present in the extracts of *R. gallicum *bv. *phaseoli *8a3 and *R. etli *12a3 is unknown. It might be that glutamate is accumulated in these strains only during the early osmostress response, as the most commonly used charge counterbalance for K^+ ^influx [4], but it is subsequently replaced by trehalose. This was previously demonstrated in *S. meliloti *by Gouffi et al [35]. On the other hand, mannosucrose and glutamate were the main osmolytes in *A. tumefaciens *10c2 grown at high salinity, whereas at low salt only mannitol was observed. Mannosucrose accumulation was found to be NaCl-dependent in *A. tumefaciens *10c2 (this study), *A tumefaciens *strains C58 and NT1 [31] and in rhizobial isolates from Acacia nodules [36], supporting the hypothesis that this compatible solute participates in alleviating osmotic stress. However, isolation and analysis of osmosensitive mutants would be necessary to prove the latter statement, and additional mechanisms involved in *A. tumefaciens *10c2 osmoadaptation cannot be ruled out.

In the tested strains, mannitol was not accumulated when glucose was used as a carbon source (Figure 4, and data not shown). On the other hand, cells grown with [1/6-^13^C]mannitol as a carbon source accumulated [1/6-^13^C]mannitol, indicating that mannitol was not synthesized *de novo *but accumulated upon transport from the external medium. Bacteria rarely synthesize mannitol as a compatible solute, but it is frequent to find it as an external osmoprotectant [4]. In general, uptake and accumulation of osmoprotectants is preferred over the synthesis of endogenous compatible solutes, as the latter is energetically more costly [37]. However, *R. tropici *CIAT 899 and *A. tumefaciens *10c2 used mannitol both as carbon source and as an osmoprotectant solute at low salinity, but mannitol was replaced by endogenous compatible solutes (i.e. trehalose or mannosucrose) when cells were exposed to hyperosmotic stress (see Figures 3 and 4). This finding may be explained by two, non-exclusive, reasons: (i) that trehalose and mannosucrose are better osmolytes than mannitol, and/or (ii) that energy-requiring systems, other than trehalose or mannosucrose synthesis, were operating at high salinity, and mannitol catabolism was enhanced in detriment of its accumulation.

The role of trehalose as a compatible solute involved in bacterial tolerance to osmotic stress has been widely demonstrated in the literature. Thus, *E. coli *[38], *S. meliloti *[12] and *B. japonicum *[13] mutants lacking the *otsA *gene for the synthesis of trehalose are osmosensitive. In another study, Alarico et al. [39] found a direct correlation between the presence of genes for trehalose synthesis (*otsA*/*otsB*) in *Thermus thermophilus *strains and their halotolerance. In this work, we found that trehalose synthesis in *R. tropici *CIAT 899 is osmoregulated (Figure 6), suggesting the involvement of trehalose in the osmotolerance of this strain. However, we could not find a direct correlation between the trehalose content of the rhizobial strains and their osmotolerance. On the contrary, trehalose levels in the less salt tolerant strains grown at 0.1 M NaCl were 10 fold-higher than those of the more salt-tolerant *R. tropici *CIAT 899 grown under the same conditions (Figure 6). Therefore, trehalose alone cannot account for the higher osmotolerance of *R tropici *CIAT 899. It is improbable that accumulation of mannitol by *R tropici *CIAT 899 conferred it a higher halotolerance, as mannitol was also accumulated by the less salt-tolerant strains. Other salt-induced responses, as modifications in the pattern of extracellular polysaccharides and lipopolysaccharides might be involved [3]. Upon transposon mutagenesis, Nogales et al [27] identified eight gene loci required for adaptation of *R tropici *CIAT 899 to high salinity. These included genes involved in regulation of gene expression, genes related to synthesis, assembly, and maturation of proteins, and genes related with cellular buildup and maintenance.

To date, three different enzymatic pathways have been described for trehalose synthesis in rhizobia (OtsAB, TreS and TreYZ; [40]). The most common two-step OtsAB pathway catalyzes the synthesis of trehalose from UDP-glucose and glucose 6-phosphate. Trehalose synthase (TreS) catalyzes the reversible conversion of maltose and trehalose. Finally, the two-step TreYZ pathway acts in the production of trehalose from a linear maltodextrin (e.g., glycogen) [32]. In this work, we showed the presence of *otsA *within the genome of the four *Rhizobium *analyzed strains, suggesting that trehalose synthesis in these strains occurs at least via OtsAB. Synthesis of trehalose from maltooligosaccharides in *R. tropici *CIAT 899 was earlier reported [41], although TreY activity could not be detected [40]. Interestingly, the phylogenetic position of OtsA from *R. gallicum *bv *phaseoli *8a3 and *R. etli *12a3 was not consistent with the 16S rDNA-based tree, suggesting the existence of lateral transfer events. Avonce et al. [32] also found inconsistencies in the topology of a proteobacterial OtsA-based tree, and suggested to be caused by either lateral gene transfer or differential loss of paralogs.

Cyclic (1→2)-β-glucans have a role in hyposmotic adaptation of the legume symbiont rhizobiaceae [8]. In *R. tropici *CIAT 899 (and probably *R. gallicum *bv. *phaseoli *8a3) cells grown at low salinity, the cyclic β-glucan was co-extracted with the cytoplasmic compatible solute pool, suggesting that high amounts of beta glucan were present in the periplasm.. As trehalose, cyclic (1→2)-β-glucans are synthesized from UDP-glucose [8]. We found that mannitol and galactose were substrates for both trehalose and the β-glucan of *R. tropici *CIAT 899. In contrast, mannose was a substrate for the β-glucan but not for trehalose.. From the above data, we conclude that *R. tropici *CIAT 899 can convert mannitol and galactose into UDP-glucose and glucose-6-phosphate, the two trehalose precursors, but it cannot transform mannose into glucose-6-phosphate. In *E. coli *and other bacteria, galactose degradation pathway I (Leloir pathway) can yield both UDP-glucose and glucose-6-phosphate [42]. Thus, a similar route might be operating in *R. tropici *CIAT 899. By using [1/6-^13^C]mannitol as a carbon source, we showed that both trehalose moieties, as well as the β-glucan units, where derived directly from mannitol. In *E. coli *and other bacteria, mannitol and mannose enter the cell via specific phosphotransferase systems so the first intracellular species are mannitol-1-phosphate and mannose-6-phosphate, respectively. In a second step, these phosphoderivatives are converted by a single dehydrogenase or isomerase reaction, respectively, into the glycolytic intermediate fructose-6-phosphate, which in turn is converted to glucose-6-phosphate by the action of a phosphoglucose isomerase [43,44]. A search in the KEGG specialized pathway database [45] showed that the genomes of *R. etli *CFN 42, *R. leguminosarum *bv. *viciae *3841, *S. meliloti *1021, *A. tumefaciens *C58, *Mesorhizobium loti *MAFF303099, *B. japonicum *USDA 110 and *Rhizobium *sp. NGR 234, among others, do not carry the *mtlA *gene encoding the specific mannitol phosphotransferase, suggesting that in the *Rhizobiaceae *mannitol do not use a phosphotransferase system to enter the cell. Instead, we found the *smoEFGK *genes encoding a sorbitol/mannitol ABC transporter, *mtlK *(encoding a mannitol 2-dehydrogenase that converts mannitol to fructose), and *xylA *(encoding a xylose isomerase that converts fructose to glucose). By analogy with these phylogenetic relatives, we suggest that in *R. tropici *mannitol could be converted into glucose via fructose. In the case of mannose, we found that the above genomes carried *manX*, encoding the phosphohistidine-sugar phosphotransferase protein, suggesting that the first intracellular species is mannose-6-phosphate. The gene *manA*, encoding the mannose-6-phosphate isomerase (isomerizing mannose-6-phosphate into fructose-6-phosphate) is present in *S. meliloti*, *Rhizobium *sp. NGR 234, *A. tumefaciens *and *B. japonicum*, but not in *R. etli*, *R. leguminosarum*, or *M. loti*. This finding suggests that the latter microorganisms, and most probably *R. tropici *CIAT 899, cannot convert mannose-6-phosphate into fructose-6-phosphate, and consequently it cannot yield glucose-6-phosphate. *R. etli*, *R. leguminosarum *and *M. loti *carried *noeK*, encoding a phosphomannomutase that converts mannose-6-phosphate to mannose-1-phosphate, and *noeJ*, encoding a mannose-1-phosphate guanylyltransferase that converts mannose-1-phosphate to GDP-mannose, a precursor for glucan biosynthesis. In addition, *R. tropici *CIAT899 carries a *noeJ*-like gene, as described by Nogales et al [27]. Again by analogy with its close relatives, we suggest that a similar pathway might be operating in *R. tropici*, explaining why this microorganism can synthesize the cyclic β-glucan from mannose, but cannot convert mannose into trehalose.

## Conclusions

The accumulation of compatible solutes is referred as one of the main mechanisms of bacterial tolerance to osmotic stress conditions such as salinity and drought. In this work, we found that all *Rhizobium *strains tested synthesized trehalose, whereas the most NaCl-tolerant strain A. *tumefaciens *10c2 synthesized mannosucrose. Whereas this finding suggests that mannosucrose might be a better compatible solute than trehalose, this would need experimental support. Despite trehalose synthesis was osmoregulated in *R. tropici *CIAT 899, our data suggest that trehalose alone cannot account for the higher osmotolerance of this strain. Thus, osmoadaptation in *R. tropici *CIAT 899 (and most soil microorganisms) is probably a complex process involving many physiological and biochemical response mechanisms, not yet fully elucidated. Although trehalose, without doubt, participates in some way to alleviate osmotic stress, there is increasing evidence that trehalose is primarily a stress metabolite designed to ensure cell survival. In fact, trehalose synthesis in *E. coli *is under the control of the general stress factor σ^S^, which is responsible for the expression of genes induced upon entry of stationary phase [38]. In *S. meliloti*, trehalose synthesis is under the control of the general stress factor RpoE2 [46], which is also necessary for desiccation resistance [47]. Thus, it may be possible that NaCl-induced synthesis of trehalose and mannosucrose in the isolated soil strains are also involved in drought tolerance. This will be investigated in a future work.

In this work, we showed the presence of *otsA *within the genome of the four studied *Rhizobium *strains, suggesting that trehalose synthesis in these strains occurs at least via OtsAB. In addition, by using [1/6-^13^C]mannitol as a carbon source, we showed that in *R. tropici *CIAT 899 both trehalose moieties, as well as the β-glucan units, where derived directly from mannitol. This finding, together with *in silico *analysis of rhizobial genomes, suggests that *R. tropici *takes up mannitol via a sorbitol/mannitol ABC transporter. Subsequently, mannitol is converted to fructose (by a mannitol 2-dehydrogenase) and the latter one into glucose, the trehalose precursor, by a xylose isomerase. In the case of mannose, the *in silico *analysis suggest that *R. tropici *incorporates it through a phosphotransferase system, yielding mannose-6-phosphate, but it cannot convert mannose-6-phosphate into fructose-6-phosphate, as it may lack the mannose-6-phosphate isomerase. This metabolic reconstruction would explain why *R. tropici *CIAT 899 cannot synthesize trehalose from mannose.

## Methods

### Bacterial strains and growth conditions

Bacterial strains used in this study were *R. gallicum *bv. *gallicum *8a3, *R. leguminosarum *bv. *phaseoli *31c3, *R. etli *12a3, *Agrobacterium *sp. 10c2 (in this work renamed as *A. tumefaciens *10c2) [23,24], and *R. tropici *CIAT 899^T ^[15]. The reference strain *R. tropici *CIAT 899^T ^belongs to the CIAT (International Center for Tropical Agriculture, Colombia) culture collection. It is able to form effective symbiosis with *P. vulgaris *and *Leucaena *trees [15] and to tolerate high temperature, low pH, and salinity [25,26]. Rhizobial strains were routinely grown in complex TY medium [48] at 28°C. For determination of salinity range and preparation of cell extracts for analysis of compatible solutes, two minimal media were used: MAS medium [49] or M79-I. M79-I is a modified M79 medium [50] in which yeast extract was substituted by 2.75% KNO_3_. The basal salinity of both M79-I and MAS was 17 mM NaCl. The osmotic strength of the media was increased by the addition of 50 to 600 mM final concentrations of NaCl. Glucose, mannitol, mannose, galactose or 1/6-^13^C-mannitol was used as carbon source at a final concentration of 20 mM. Growth was monitored by measuring the optical density at O.D._600 _of the cultures with a Perkin Elmer Lamda 25 UV/Vis spectrophotometer.

### Preparation of cell extracts, NMR spectroscopy and Mass spectrometry

Rhizobial strains were grown in 200 ml of M79-I or MAS minimal media up to late exponential/early stationary phase phase of growth. Carbon source and NaCl concentrations used varied according to the strain. Extraction of endogenous compatible solutes was performed as described by García-Estepa et al. [51]. For ^1^H- and ^13^C-nuclear magnetic resonance (NMR) spectroscopy, dried extracts were resuspended in D_2_O (0.5 ml). NMR spectra were recorded at 25°C on a Bruker AV500 spectrometer at 500 MHz for ^1^H-NMR and 125 MHz for ^13^C-NMR. The chemical shifts are reported in ppm on the *δ *scale relative to tetramethylsilane. Signals corresponding to trehalose, glutamate, mannosucrose, and mannitol were confirmed by comparison with previously ^1^H- and ^13^C-NMR spectra of pure compounds or published chemical shift values [31]. Signals in the NMR spectra of the unknown sugar observed in *R. tropici *CIAT 899 extracts (later on identified as a β-glucan) were assigned by using a suite of COSY (correlated spectroscopy), 1 D NOESY (nuclear Overhauser effect spectroscopy), HSQC (heteronuclear single-quantum coherence), and HMBC (heteronuclear single-quantum coherence) experiments. The final cyclic (1→2)-β-glucan structure was determined by Mass spectrometry by using a Applied Biosystems QTRAP LC/MS/MS system (Foster City, USA) consisting of an hybrid triple quadrupole linear ion trap (QqQ_LIT_) mass spectrometer equipped with an electros pray ion source (Turbo IonSpray). This structure was later confirmed by literature data [34].

### Determination of protein content

To estimate total cell proteins, each rhizobial strain was grown at 28°C in its optimal minimal medium until late exponential/early stationary phase. The same culture was used for determination of both trehalose and protein content. Cell protein content was determined by triplicate by using the "Test-tube procedure" of the bicinchoninic acid (BCA) protein assay kit (Pierce). Cell suspensions (1 ml) were centrifuged at 13,000 rpm for 4 min and the supernatant was removed. Cell pellets were dried overnight at 100°C and resuspended in 1 ml of demineralized water by shaking at room temperature for 30 min. After addition of 2 ml of the BCA reagent to 100 μl samples, incubation was done at 37°C (standard protocol, working range of 20-2,000 μg protein ml^-^1) or 60°C (enhanced protocol, working range of 5-250 μg protein ml^-1^) for 30 min. Then, absorption of samples was measured at 562 nm in a Perkin Elmer Lambda 25 UV/Vis spectrophotometer and compared to protein standards containing bovine serum albumin in a concentration range of 0-600 μg ml^-1^.

### Extraction and determination of intracellular trehalose content

Trehalose determination was performed basically as described by Blázquez et al. [52] by the following procedure. Cell pellets from 15 ml of early stationary phase cultures in their optimal minimal medium were washed with isotonic carbon-free medium and resuspended in 1 ml of the same medium. Cells were lysed by 30 min incubation at 95°C and, after centrifugation, trehalose was assayed in a 200 μl total volume reaction containing 100 μl of the supernatant, 90 μl of 25 mM sodium acetate buffer (pH 5.6) and 0.02 U of commercial trehalase (Sigma). For each culture sample, endogenous glucose content was monitored by performing a parallel reaction in which trehalase was substituted by water. After overnight incubation at 37°C, glucose released by trehalose hydrolysis was determined on 150 μl of the previous reaction by addition of 150 μl of a glucose oxidase/peroxidase mixture (0.66 mg ml-^1^) *Aspergillus niger *glucose oxidase and 0.25 mg ml-^1 ^horseradish peroxidase in 0.5 M phosphate buffer, pH 6.0 (Sigma) and 50 μl of 2.33 mg ml-^1 ^o-toluidine. After 30 min incubation at 37°C, 1.5 ml of water was added to the samples and absorption was measured at 420 nm in a Perkin Elmer Lambda 25 UV/Vis spectrophotometer and compared to glucose standards in a concentration range of 0-300 μg ml^-1^. Finally, trehalose content was inferred from the glucose content by performing a standard curve with commercial trehalose (Sigma) ranging from 1 to 5 mM. Trehalose concentration was expressed as μmol mg protein^-1^.

### Isolation of the *otsA *and 16S rRNA genes

Total DNA was isolated by using the CTAB method [53]. Amplification of about 1-kb of the *otsA *gene from *R. gallicum *bv. *phaseoli *8a3, *R. leguminosarum *bv. *phaseoli *31c3, and *R. etli *12a3 was performed by using the primers OTA1: 5'-ATC TGG ATG GGA TGG TCG GGA-3' and OTA2: 5'-GAC ATA TTC CTT GGC AAC GAG GTT-3'. For strain CIAT 899, o*tsA *was amplified by using the degenerated primers: OTAS1: 5'-CAT CTG GAT GGG (CT)TG GTC GG-3' and OTAS2: 5'-GGC GAC ATA TTC CTT GGC (GC)AC (GC)AG GTT-3'. The amplification protocol consisted of the following steps: initial denaturation at 94°C for 5 min followed by 30 cycles of denaturation (45 seconds at 94°C), annealing (45 seconds at 58°C), extension (1 min at 72°C), and a final extension step at 72°C for 10 min. Sequencing of the *otsA *genes was performed by the company Newbiotechnics (NBT, Seville, Spain). PCR amplifications of the complete 16S rRNA genes were carried out as previously described [54]. The PCR products were used as templates in sequencing reactions with internal primers to get partial sequences of the 16S rRNA gene (NBT, Seville, Spain). The resulting overlapping sequences were analyzed by using the ChromasPro software (version 1.34) to assemble the complete 16S rRNA gene of each strain.

### Phylogenetic analysis

The 16S rRNA gene and OtsA protein sequences were used as queries for BLAST searches at the NCBI (National Center for Biotechnology Information) web server http://www.ncbi.nlm.nih.gov/. Homologous and validated (for 16S rRNA) sequences showing a high degree of similarity were included in the phylogenetic analyses. 16S rRNA-based and OtsA-based phylogenetic analyses were conducted by using the MEGA 4 software [55]. Nucleotide (16SrRNA) alignments were constructed with Clustal W (1.6). The tree was constructed by using the neighbor-joining method [56] and the evolutionary distances were computed using the two-parameter method [57]. The rate variation among sites was modeled with a gamma distribution (shape parameter = 0.25) and all positions containing alignment gaps and missing data were eliminated only in pairwise sequence comparisons. The robustness of the tree branches was assessed by performing bootstrap analysis of the neighbor-joining data based on 1000 resamplings [58]. There were a total of 1469 positions in the final dataset. The partial OtsA protein-coding sequences were aligned with Clustal W (1.6) using a BLOSUM62 matrix and manually edited. The phylogenetic tree was inferred using the neighbor-joining method and the evolutionary distances were computed using the Poisson correction method. The rate variation among sites was modeled with a gamma distribution (shape parameter = 1) and all the positions containing gaps and missing data were eliminated from the dataset obtaining a total of 287 positions. The robustness of the tree branches was assessed by performing bootstrap analysis of the neighbor-joining data based on 1000 resamplings.

### Nucleotide sequence accession numbers

The 16S rRNA and *otsA *gene sequences generated in this study correspond to *R. leguminosarum *bv. *phaseoli *31c3 16S rDNA [EMBL:FN433080], *R. gallicum *bv. *phaseoli *8a3 16S rDNA [EMBL:FN433081], *A. tumefaciens *10c2 16S rDNA [EMBL:FN433082], *R. etli *12a3 16S rDNA [EMBL:FN43308], *R. etli *12a3 *otsA *[EMBL:FN433084], *R. leguminosarum *bv. *phaseoli *31c3 *otsA *[EMBL:FN433085], *R. gallicum *bv. *phaseoli *8a3 *otsA *[EMBL:FN433086], and *R. tropici *CIAT 899 *otsA *[EMBL:FN433087].

## Abbreviations

NMR: nuclear magnetic resonante; MHz: megahertz; TMS: tretramethylsilane; COSY: correlated spectroscopy; HSQC: heteronuclear single-quantum coherente; HMBC: heteronuclear multiple-bond correlation; NOESY: nuclear Overhauser effect spectroscopy; MS: mass spectrometry

## Authors' contributions

CFA and TBH performed the majority of the experiments, and participated in bioinformatic analysis. FIG contributed to NMR analysis, MA performed the phylogenetic analysis. MRB performed some growth experiments and trehalose determination, JJN participated in bioinformatic analysis and figure preparation. MEA and CV conceived the study, participated in the design, coordination, bioinformatic analysis, and writing of the manuscript. All authors have read and approved the final manuscript.
